# S100A8 is a prognostic signature and associated with immune response in diffuse large B-cell lymphoma

**DOI:** 10.3389/fonc.2024.1344669

**Published:** 2024-02-01

**Authors:** Qi Lin, Jianlin Su, Yuanyuan Fang, Zhihao Zhong, Jie Chen, Chaofeng Zhang

**Affiliations:** ^1^ Department of Pharmacy, The Affiliated Hospital of Putian University, Putian, Fujian, China; ^2^ Pharmaceutical and Medical Technology College, Putian University, Putian, Fujian, China; ^3^ Department of Hematology and Rheumatology, the Affiliated Hospital of Putian University, Putian, Fujian, China

**Keywords:** diffuse large B cell lymphoma, S100A8, immune response, immunotherapy, prognosis

## Abstract

**Background:**

S100A8, a calcium-binding protein belonging to the S100 family, is involved in immune responses and multiple tumor pathogens. Diffuse large B-cell lymphoma (DLBCL) is one of the most common types of B-cell lymphoma and remains incurable in 40% of patients. However, the role of S100A8 and its regulation of the immune response in DLBCL remain unclear.

**Methods:**

The differential expression of S100A8 was identified via the GEO and TCGA databases. The prognostic role of S100A8 in DLBCL was calculated using the Kaplan-Meier curve. The function enrichment of differentially expressed genes (DEGs) was explored through GO, KEGG, GSEA, and PPI analysis. In our cohort, the expression of S100A8 was verified. Meanwhile, the biological function of S100A8 was applied after the inhibition of S100A8 in an *in vitro* experiment. The association between S100A8 and immune cell infiltration and treatment response in DLBCL was analyzed.

**Results:**

S100A8 was significantly overexpressed and related to a poor prognosis in DLBCL patients. Function enrichment analysis revealed that DEGs were mainly enriched in the IL-17 signaling pathway. Our cohort also verified this point. *In vitro* experiments suggested that inhibition of S100A8 should promote cell apoptosis and suppress tumor growth. Single-cell RNA sequence analysis indicated that S100A8 might be associated with features of the tumor microenvironment (TME), and immune infiltration analyses discovered that S100A8 expression was involved in TME. In terms of drug screening, we predicted that many drugs were associated with preferable sensitivity.

**Conclusion:**

Elevated S100A8 expression is associated with a poor prognosis and immune infiltration in DLBCL. Inhibition of S100A8 could promote cell apoptosis and suppress tumor growth. Meanwhile, S100A8 has the potential to be a promising immunotherapeutic target for patients with DLBCL.

## Introduction

Globally, diffuse large B-cell lymphoma (DLBCL) is the most common type of lymphomas in adults, which is highly heterogeneous and accounts for the majority of newly diagnosed non-Hodgkin lymphoma (NHL) cases (1–4). With R-CHOP (rituximab, cyclophosphamide, doxorubicin, vincristine, and prednisone) treatment, about 65% of DLBCL cases can be cured (5). However, 40-45% of DLBCL patients are still refractory or relapsed, with a poor prognosis (6, 7). Recently, the molecular heterogeneity of DLBCL has undergone in-depth exploration with the utilization of genomics and transcriptomics over the past two decades; nonetheless, the molecular mechanism of DLBCL has been under investigation. The tumor microenvironment (TME) has been closely related to the origination, progression, and metastasis of DLBCL (8, 9). Different components of the TME can promote tumor development and angiogenesis, which have been recognized as tumor development and immune escape (10, 11). Alterations in genetic signatures in the TME are presumed to play a significant role in prognostic significance, tumor progression, and treatment outcome (8, 12, 13). Hence, explorations of the DLBCL pathological mechanisms and new regulators are necessary.

S100A8 (also called MRP-8 and Calgranulin A), which is a calcium-binding protein belonging to the S100 protein family with a low molecular weight (14–16), plays crucial roles in normal biological processes and pathological conditions (15, 17). S100A8 that is located in the cytosol of resting phagocytes can be released by activated phagocytes and be followed by two independent translocation pathways (15, 18). S100A8 could exhibit effects in inflammation, immune response, and cancer development, but its biological functions are complex. As previously reported, overexpression of S100A8 is associated with tumorigenesis and poor prognosis in various cancers (16, 19). Wang H et al. reported that S100A8 and S100A9 were highly expressed in glioma patients and played a marker correlated with survival and prognosis (20). A significantly higher serum level of S100A8 in pancreatic cancer patients with cachexia patients appeared, indicating S100A8 can play an atrophic role in pathogenic development (21). Several studies suggested S100A8 played a role in mediating drug resistance in multiple hematological malignancies (22, 23). Aldebert et al. indicated that overexpression of S100A8/S100A9 in B leukemic cells introduced an increase in myeloid subpopulations and suggested the relapse of acute lymphoid leukemia (ALL) through remodeling of the TME (22). A high transcription level of S100A8 exhibited poor survival in acute myeloid leukemia (AML) patients (24), and a lower apoptosis rate was exhibited in AML cells transfected with S100A8 (24). Moreover, inhibition of S100A8 could improve chemotherapy sensitivity by reducing autophagy in B-cell lymphoma cells with strong drug resistance (25). Simultaneously, S100A8/S100A9 activation facilitates leukocyte recruitment, angiogenesis, and tumor migration, along with the enhancement of some genes, including Cxcl1, Ccl5 and Ccl7, Slc39a10, Lcn2, Zc3h12a, Enpp2 and other genes (26, 27). Generally, the role of S100A8 in multiple tumors is highlighted as a promotor of tumor invasion, TME formation, and drug resistance (14, 16, 28), however, there has been no direct report on relationship between S100A8 and DLBCL.

In this study, we explored the clinical application value of S100A8 in DLBCL using public databases and our cohort and clarified its potential mechanisms. The biological function of S100A8 in DLBCL *in vitro* experiments was comprehensively analyzed. Next, we investigated the correlation between S100A8 and immune infiltration in DLBCL, focusing on the probable therapeutic compounds. Thus, the results of this study provided further knowledge of S100A8 in DLBCL and highlighted that S100A8 should be a new therapeutic target for DLBCL.

## Materials and methods

### Data obtaining and processing

R programming was applied for integrating, analyzing, and visualizing the data. The transcriptome profile and clinical data of DLBCL were collected from the cancer genome atlas (TCGA, https://portal.gdc.cancer.gov/) and the Gene Expression Omnibus (GEO, https://www.ncbi.nlm.nih.gov/geo/), including GSE83632 (29) (n = 163), GSE56315 (30) (n = 88), GSE87371 (31) (n = 223), NCICCR_DLBCL (1) (n = 481), and GSE31312 (32) (n = 498). Differential expression of *S100A8* mRNA in DLBCL tissues and normal controls (NCs) was compared using limma package (33), and the performance of S100A8 in DLBCL was analyzed based on the receiver operating characteristic (ROC) curve. The protein levels of S100A8 were investigated through The Human Protein Altas (HPA, https://www.proteinatlas.org). The value of S100A8 in predicting DLBCL patient survival was analyzed utilizing the log-rank test and Cox regression model using survival and survminer packages.

### Differentially expressed gene enrichment analysis

The differentially expressed genes (DEGs) of high and low *S100A8* expressions divided by median were screened using limma package (33) in training and validation datasets and defined as significantly DEGs according to |logFC| > 1 and adjusted *P* value < 0.05. The overlapping DEGs between training and validation datasets were identified using ggvenn package, and inputted into the Database for Annotation, Visualization, and Integrated Discovery (DAVID) (34) where Gene Ontology (GO) and Kyoto Encyclopedia of Genes and Genome (KEGG) analysis (35) were performed. Gene set enrichment analysis (GSEA) was applied to analyze signaling pathways enriched for the overlapping DEGs in both training and validation datasets through the WEB-based Gene SeT AnaLysis Toolkit (WebGestalt, http://www.webgestalt.org/#) (36). The DEGs were inputted to the Search Tool for the Retrieval of Interacting Genes (STRING, http://string-db.org) (37), and protein-protein interaction (PPI) analysis was further investigated.

### Clinical specimens collection

In this study, the formalin-fixed paraffin-embedded (FFPE) lymph node tissue blocks of 39 patients, including 25 DLBCL patients and 14 reactive hyperplasia (RHL) patients, were retrospectively enrolled from the Department of Pathology in our institution; the clinical characteristics were provided in **Supplementary Table S1** and stored at room temperature. This study was authorized by the Affiliated Hospital of Putian University’s Ethics Committee (ID: 2023075).

### Immunofluorescence and immunohistochemistry staining

Immunofluorescence (IF), as an important immunochemical technique, can allow the detection and localization of a wide variety of antigens in different types of tissues. The FFPE blocks collected were sliced (4 μm sections) and deparaffinized in xylene, rehydrated in alcohol, and then washed in distilled water. For antigen retrieval, the sections were microwaved in citrate buffer and blocked using a protein block. The sections were incubated with rabbit anti-S100A8 polyclonal (Beyotime, China) and mouse anti-IL17F monoclonal (Santa Cruz, USA). Subsequently, secondary antibodies FITC-labeled goat anti-rabbit IgG (Beyotime, China) and cy3-labeled goat anti-mouse IgG (Beyotime, China) were added and incubated at room temperature for visualization. The sections were counter-stained with DAPI (Beyotime, China), and then images were captured by an automatic quantitative pathology imaging system (Vectra Polaris, PerkinElmer, Waltham, MA). For performing immunohistochemistry (IHC), the sections were incubated with the mouse anti-S100A8 monoclonal (Immunoway, China) overnight at 4°C, followed by HRP-labeled goat anti-mouse IgG(H+L) (Beyotime, China) treatment at room temperature for 2 hours. Third, 3,3-diaminobenzidine (DAB, Beyotime, China) was used for visualization, and the sections were imaged by a DM1L inverted microscope (Leica, Germany).

### Cell culture and treatment

The three cell lines were enrolled in this study, including one human normal B-cell line (GM12878, BeNa, China) and the DLBCL cell lines SUDHL2 (a gift from Southeast University) and SUDHL4 (Meisen, China). These cell lines were cultured in RPMI-1640 (Biosharp, China) supplemented with 10% fetal bovine serum (FBS, Gibco, US), 1% streptomycin, and penicillin (Gibco, US), and incubated at 37°C in a 5% CO_2_ incubator (1300 series A2, Thermo Fisher, US). Differential concentrations of Paquinimod (ABR-215757, MedChemExpress, US) were applied to DLBCL cell lines for 24 h.

### Quantitative real-time polymerase chain reaction

Total RNA of cell lines was isolated using TRIzol reagent (Invitrogen, US), and then HiScript Q RT SuperMix for qPCR (Vazyme, China) was used to transcribe the RNA into cDNA. The quantitative real-time polymerase chain reaction (qRT-PCR) was conducted on the CFX Connect Real-Time PCR Detection System (BioRad, US) with an HQ SYBR qPCR Mix (without ROX) (Zomen, China). The S100A8 expressions were quantified with the 2^−ΔΔCt^ method, and β-actin expression was used as an endogenous control. The primer sequences are shown in **Supplementary Table S1**.

### Western blotting analysis

The protein expression of S100A8 in cell lines was validated through WB analysis. First, the total protein was extracted using RIPA buffer (Beyotime, China) with 1% PMSF (Beyotime, China), and the concentration of protein was measured using a BCA protein assay kit (Beyotime, China). Then, the extracted protein was loaded onto a 12.5% SDS-PAGE gel (Meilunbio, China) and transferred onto polyvinylidene fluoride (PVDF) membranes. The membranes were incubated with primary antibodies [anti-S100A8 polyclonal (Immunoway, China), anti-BCL2 polyclonal (Beyotime, China), anti-Bax polyclonal (Beyotime, China), anti-Caspase-3 polyclonal (Beyotime, China), and anti-β-actin polyclonal (Beyotime, China)] at 4°C overnight. After the membranes were incubated with HRP-labeled goat anti-rabbit IgG (Beyotime, China), At room temperature, the level of protein was detected using BeyoECL Plus (Beyotime, China) and quantified using Fiji (version 2.9, fiji.sc).

### Cell inhibition detection

A density of 5 × 10^4^ DLBCL cell lines were seeded in the 96-well plates in 200 μL culture medium. After exposure to Paquinimod for 24 h, 20 μL of CCK-8 reagent (Biosharp, China) was added into each well, and the absorbance (OD) of every well was measured by a Thermo Multiskan FC enzyme reader (Thermo Fisher, USA) after incubation for 2 h. Each concentration accounted for in the control was presented as cell inhibition, and the half maximal inhibitory concentration (IC50) was calculated using R programming. Meanwhile, cell apoptosis was conducted according to the manufacturer’s protocols using an Annexin V–FITC/propidium iodide (PI) Apoptosis Detection Kit (Zomanbio, China), and then the cells were detected by a FacsCalib flow cytometer (BD Biosciences, USA).

### Single-cell RNA sequencing analysis

The single-cell RNA sequencing (scRNA-seq) was extracted from the GSE182434 (38), the Seurat package (39) to normalize and scale the expression matrices. The top 2000 most highly variable genes were selected for principal component analysis. An Uniform Manifold Approximation and Projection (UMAP) algorithm was used to visualize cells. Then, the expression of S100A8 in differential cell types was annotated, and the enrichment was explored.

### TME and immune cell infiltration

The connection between immune infiltrates and S100A8 expression in TCGA_DLBC was explored through the TIMER2 website (40) (http://timer.cistrome.org). The S100A8-enriched immune cells were selected, and CIBERSORT, CIBERSORT-ABS, EPIC, MCPcounter, quanTIseq, TIDE, TIMER, and xCell algorithms were utilized to estimate immune infiltration. The Spearman’s rank correlation coefficient (rho) values were provided. Next, for identifying the potential connection between S100A8 and TME features, the estimate package (41) was applied to calculate the immune and stromal scores on both training and validation datasets using single sample gene set enrichment analysis (ssGSEA). Next, the correlation between the status of S100A8 and immune cell infiltration was explored through the Cell-type Identification by Estimating Relative Subsets of RNA Transcripts (CIBERSORTx, http://cibersort.stanford.edu/). Also, immunedeconv package (42) was applied to analyze the immune cell fractions. It seemed that the interaction between angiogenesis and TME is an important promoter of tumor growth, invasion, and metastases (8, 43). The angiogenesis-related genes (ARG) were obtained from GeneCards (https://www.genecards.org), and the correlation between the expression of S100A8 and ARGs was investigated.

### Drug sensitivity evaluation

Checkpoint targets play a key role in tumor immunotherapy (44). We identified the correlation between S100A8 and checkpoint targets, which was obtained from the checkpoint therapeutic target database (CKTTD) (44). To evaluate the correlation between *S100A8* expression and anticancer drug activity, the RNA expression data (RNA: RNA-seq) and drug data (compound activity: DTP NCI-60) were obtained from the CellMiner database (www.discover.nci.nih.gov) (45). Next, for predicting the drug sensitivity based on the heterogenicity of tumor samples, the oncoPredict package (46) was used to estimate the half-maximal inhibitory concentration (IC50) of cancer cell lines to build a predictive model to determine drug sensitivity in DLBCL. The potential anti-tumor compounds were screened based on the drug response data in the Genomics of Drug Sensitivity in Cancer (GDSC) database (https://www.cancerrxgene.org/), which had been analyzed to determine the relationship between *S100A8* expression and sensitivity to chemotherapy and targeted drugs (*P* < 0.01).

### Statistical analysis

All data analysis was performed in R programming (version 4.3.1) and visualized using the ggplot2 package. *P* < 0.05 was considered statistically significant.

## Results

### Elevated S100A8 expression indicated poor prognosis in public DLBCL datasets

**Figure 1:**
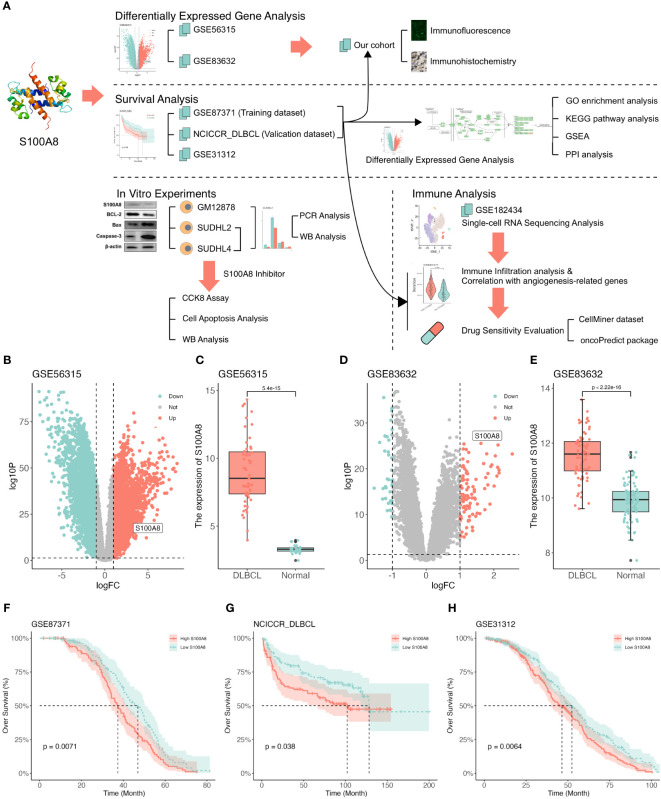
The upregulated expression of S100A8 was associated with poor survival in DLBCL. **(A)** Study flowchart. **(B)** The volcano plot based on GSE56315 datasets, S100A8 was located in the area of upregulation, adjust *P* value < 0.05 and |log(fold change)| < 1. **(C)** S100A8 expression in DLBCL samples (n = 55) compared with human healthy tonsils (n = 33) based on GSE56315 dataset. **(D)** The volcano plot based on GSE83632 datasets, S100A8 was located in the area of upregulation, adjust *P* value < 0.05 and |log(fold change)| < 1. **(E)** S100A8 expression in DLBCL whole blood samples (n = 76) compared with healthy controls (n = 87) based on GSE83632 dataset. The KM curves showed OS **(F–H)** excluded OS time less than one month in GSE87371 (n = 216), NCICCR_DLBCL (n = 228), and GSE31312 (n = 466). Diffuse large B-cell lymphoma; GSE, Gene Expression Omnibus Series; KM, Kaplan–Meier; OS, over survival.

The flow of this study is depicted in **Figure 1A**. Based on the TIMER2 website (40), the S100A8 expression in all TCGA tumors was explored (**Supplementary Figure S1A**). Next, the GSE83632 (29) (training dataset) and GSE56315 (30) (validation dataset) were used to identify DEGs between DLBCL and NCs using limma package (33) (**Figures 1B, D**), and the dramatically upregulated *S100A8* expression was shown (*P*<0.05) in **Figures 1C, E**. The area under the curve (AUC) of the ROC curve was 1 (**Supplementary Figure S1B**) and 0.928 (95% confidence interval (CI): 0.889-0.967, **Supplementary Figure S1C**), suggesting that *S100A8* had a good ability of distinguishing DLBCL from NCs. The protein levels of S100A8 in malignant lymphomas were higher than in normal lymph nodes (**Supplementary Figure S1D**). We further investigated the prognostic impact of *S100A8* on DLBCL patients. The elevated *S100A8* expression was closely related to poor overall survival (OS) in the GSE87371 (31) (HR = 1.505, 95% CI: 1.108–2.044, *P* = 0.007, **Figure 1F**), NCICCR_DLBCL (1) (HR = 1.544, 95% CI: 1.025–2.326, *P* = 0.038, **Figure 1G**), and GSE31312 (32) (HR = 1.362, 95% CI: 1.082–1.714, *P* = 0.006, **Figure 1H**). Poor progression-free survival (PFS) was linked to increased *S100A8* in the GSE87371 (31) (HR = 1.407, 95% CI: 1.018–1.944, *P* = 0.033, **Supplementary Figure S1E**) and the GSE31312 (32) (HR = 1.338, 95% CI: 1.061–1.688, *P* = 0.011, **Supplementary Figure S1F**). Meanwhile, high expression of S100A8 was seemed to be associated to poor OS in DLBCL patients with high international prognostic index (IPI, score > 2) between GSE87371 (HR = 1.764, 95% CI: 1.058–2.944, *P* = 0.021, **Supplementary Figure S1G**) and NCICCR_DLBCL (HR = 1.768, 95% CI: 0.873–3.580, *P* = 0.082, **Supplementary Figure S1H**).

### Exploration of DEGs’ biological function

**Figure 2:**
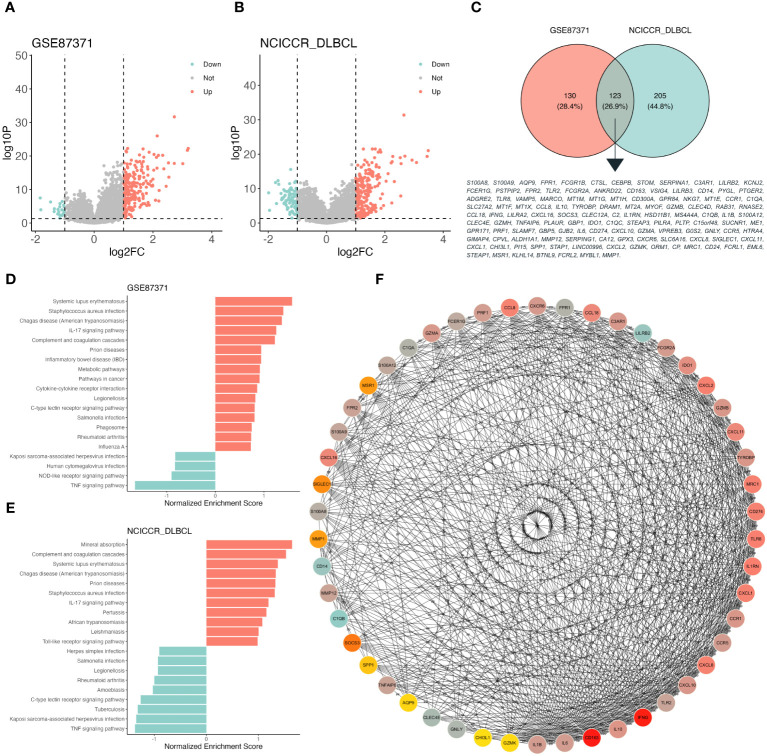
Function enrichment analysis of different S100A8 expression level in DLBCL. **(A)** The volcano plot based on high- and low- S100A8 expression group divided into median S100A8 value in GSE87371 dataset, adjust *P* value < 0.05 and |log(fold change)| < 1. **(B)** The volcano plot based on high- and low- S100A8 expression group divided into median S100A8 value in NCICCR_DLBCL dataset, adjust *P* value < 0.05 and |log(fold change)| < 1. **(C)** DEGs extracted from GSE87371 and NCICCR_DLBCL datasets, there were 123 genes enrolled. **(D, E)** Identification of S100A8-related signaling pathways by GSEA in GSE87371 and NCICCR_DLBCL datasets. **(F)** The connection between the top 50 DEGs was explored through Cytoscape software (version 3.9.1) using the CytoHubba plugin based on the MCC score. DEG, Differentially expressed gene; DLBCL, Diffuse large B-cell lymphoma; GSE, Gene Expression Omnibus Series; PPI, protein-protein interaction.

The DEGs of high and low S100A8 expression based on the median expression value were identified in the training (GSE87371, 232 upregulated and 21 downregulated genes, **Figure 2A**) and validation (NCICCR_DLBCL, 236 upregulated and 92 downregulated genes, **Figure 2B**) datasets. The 123 overlapping genes were classified through a venn plot (**Figure 2C**). The biological processes (BP) of these genes were enriched by inflammatory response, immune response, and chemotaxis. The main enrichment cellular components (CC) were plasma membranes, extracelluar regions, and extracelluar space. Meanwhile, molecular function (MF) was associated with transmembrane signaling receptor activity, signaling receptor activity, and serine-type endopeptidase activity (**Supplementary Figure S2A**). The KEGG pathway analysis indicated that DEGs were mainly involved in the IL-17 signaling pathway, viral protein interaction with cytokine and cytokine receptor, Pertussis, and Cytokine-cytokine receptor interaction (**Supplementary Figure S2B**). GSEA analysis of the training and validation datasets by WebGestalt found that DEGs could activate the IL-17 signaling pathway, complement and coagulation cascades, and inhibit the TNF signaling pathway (**Figures 2D, E**). As shown in **Figure 2F**, the connection between the top 50 DEGs was explored through Cytoscape software (version 3.9.1) using the CytoHubba plugin based on the MCC score. The PPI plot of these DEGs was drawn through STRING (**Supplementary Figure S2C**).

### Validation of S100A8 expression in our cohort

**Figure 3:**
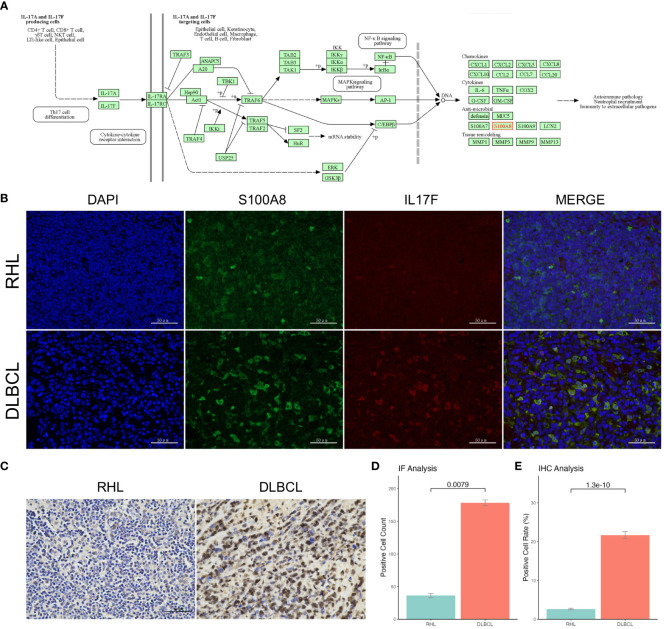
The upregulated S100A8 expression in our cohort. **(A)** S100A8 might be mainly involved in the IL-17 signaling pathway obtained from KEGG. **(B)** The IF assay was staining with rabbit anti-S100A8 polyclonal (Beyotime, China) and mouse anti-IL17F monoclonal (Santa Cruz, USA), the green fluorescence represented the expression of S100A8, and red indicated the expression of IL17F. **(D)** The S100A8 expression in DLBCL was higher than that in RHL through IF assay. **(C)** the IHC assay showed overexpression of S100A8 in DLBCL. **(E)** The S100A8 expression in DLBCL was higher than that in RHL through IHC assay. DLBCL, Diffuse large B-cell lymphoma; IF, Immunofluorescence; IHC, Immunohistochemistry.

S100A8 might be mainly involved in the IL-17 signaling pathway (**Figure 3A**), which was identified by GSEA analysis. In order to verify the role of the IL-17 signaling pathway in DLBCL patients, we collected 25 DLBCL patients and 14 RHL patients in our institution. The clinical characteristics are provided in **Supplementary Table S1**. The IF assay was performed and shown in **Figure 3B**; the green fluorescence represented the expression of S100A8, and red indicated the expression of IL17F. The results showed S100A8 expression in DLBCL was higher than that in RHL (*P* < 0.05, **Figure 3D**). Next, the IHC assay also suggested overexpression of S100A8 in DLBCL (*P* < 0.05, **Figures 3C, E**).

### Inhibition of S100A8 mediated cell apoptosis

**Figure 4:**
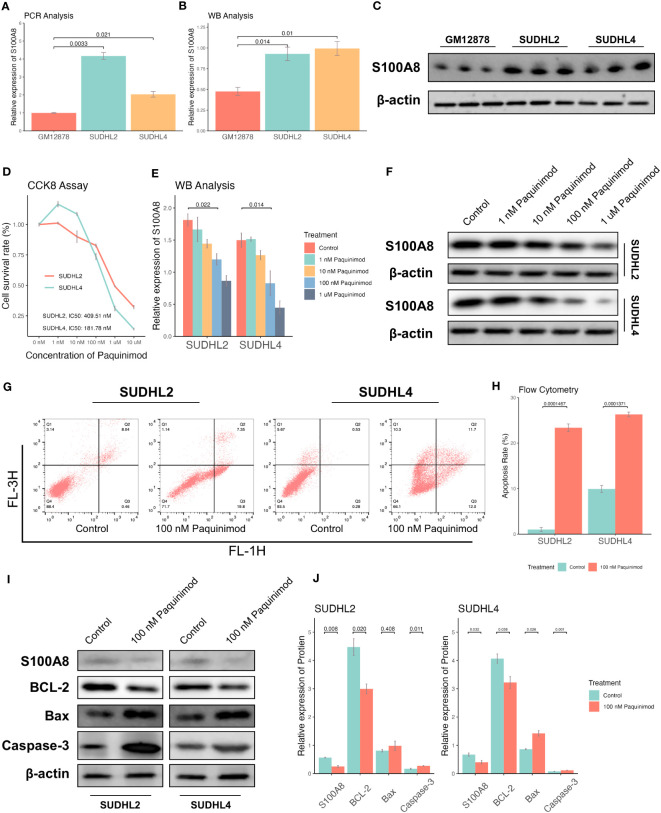
Inhibition of S100A8 promoted the cell apoptosis and suppressed tumor growth. The expression of S100A8 in different B lymphocyte cell lines, including GM12878, SUDHL2, and SUDHL4. A qRT-PCR analysis **(A)**, and a WB analysis **(B, C)**. **(D)** The cell survival rates of SUDHL2 and SUDHL4 cell lines declined by differential concentrations (control, 1 nM, 10 nM, 100 nM, 1 μM, 10 μM) of Paquinimod treatment with dose-dependent manner, and IC50 of Paquinimod treated to SUDHL2 and SUDHL4 was 409.51 nM and 181.78 nM respectively. **(E, F)** The protein expression of S100A8 declined by differential concentrations (control, 1 nM, 10 nM, 100 nM, 1 μM) of Paquinimod treatment with dose-dependent manner. **(G)** Apoptosis of SUDHL2 and SUDHL4 cells were measured by flow cytometry. **(H)** Quantitative analysis of ratio of apoptosis. **(I, J)** The protein expression of S100A8 and BCL-2 declined by 100 nM of Paquinimod treatment, as well as the expression of Bax and Caspase-3 increased. IC50, half maximal inhibitory concentration; qRT-PCR, real-time reverse transcription-PCR; WB, western Boltting.

Based on the above bioinformatics results, S100A8 is mainly involved into IL-17 signaling pathway, which can intersect with apoptosis (47, 48). To validate the potential process of cell apoptosis S100A8 mediated, We demonstrated the role of S100A8 in DLBCL cells. First, the protein level of S100A8 was decreased in SUDHL2 and SUDHL4 cell lines comparison with GM12878 cell line using qPR-PCR analysis (**Figure 4A**) and WB analysis (**Figures 4B, C**). Paquinimod, a specific inhibitor of S100A8, could participate in regulation of inflammation and immune responses (49, 50). While S100A8 inhibition with differential concentration of Paquinimod in a dose-dependent manner, the IC50 of Paquinimod treated to SUDHL2 and SUDHL4 was 409.51 nM and 181.78 nM respectively using drc package (51) (**Figure 4D**). Meanwhile, the protein levels of S100A8 could inhibit by differential concentration of Paquinimod (**Figures 4E, F**). After the treatment of 100 nM Paquinimod, cell apoptosis rates of SUDHL2 and SUDHL4 increased (**Figure 4G, H**), the protein levels of S100A8, BCL2 were significantly decreased, while Bax and Caspase-3 levels were increased (**Figure 4I, J**). These findings indicated that inhibition of S100A8 could augment cell apoptosis and induce cell death of DLBCL cell lines.

### Generation of a single-cell Atlas for DLBCL

**Figure 5:**
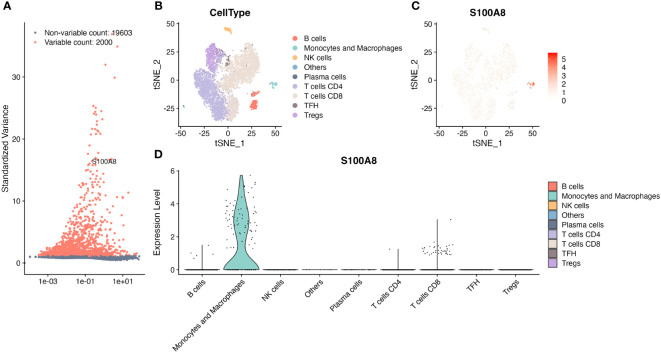
The single-cell RNA sequencing analysis in GSE182434. **(A)** S100A8 was one of top 2000 highly variable genes. **(B)** Identification of major cell types. **(C)** The tSNE plot of S100A8 from four DLBCL in GSE182434 dataset. **(D)** The violin plot showed S100A8 mainly expressed at different levels in macrophages, CD8 T cells, and B cells. DLBCL, Diffuse large B-cell lymphoma; GSE, Gene Expression Omnibus Series.

The single-cell analysis revealed that S100A8 was a highly variable gene (**Figure 5A**). We found that S100A8 was mainly expressed at different levels in macrophages, CD8 T cells, and B cells, specifically macrophages (**Figures 5B–D**), and S100A8 expression was highest in macrophages.

### Correlation analysis of S100A8 with TME

**Figure 6:**
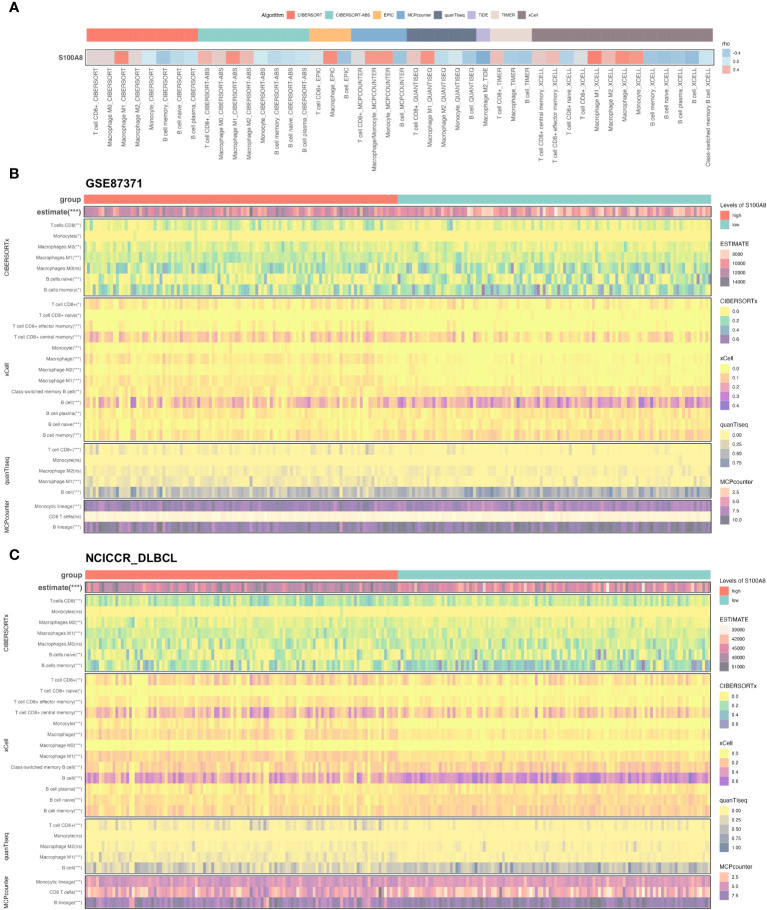
The immune infiltration associations about S100A8 in DLBCL. **(A)** The correlation between S100A8 and three cell types (macrophages, CD8 T cells, and B cells) through the TIMER2 website (http://timer.cistrome.org). **(B, C)** The Heatmap showing the connection between S100A8 and three cell types (macrophages, CD8 T cells, and B cells) using estimate, CIBERSORTx, xCell, quanTIseq, MCPcounter algorithms in GSE87371 and NCICCR_DLBCL datasets. CIBERSORT, cell type identification by estimating relative subsets of RNA transcripts; DLBCL, Diffuse large B-cell lymphoma; GSE, Gene Expression Omnibus Series; MCP-counter, Microenvironment Cell Populations-counter; quanTIseq, quantification of the Tumor Immune contexture from human RNA-seq data; xCell, digitally portraying the tissue cellular heterogeneity landscape. *** P < 0.001, ** P < 0.01, * P < 0.05, ns, not signifcance.

The dynamic interaction between tumor cells and the surrounding TME is of paramount importance in the processes of tumor initiation, development, metastasis, and therapeutic response (9, 10, 52). Based on the result of scRNA-seq analysis, we found a statistically significant negative association between S100A8 expression and B cells, also S100A8 was a positive correlation with macrophages and CD8 T cells (**Figure 6A**) through the TIMER2 website (40). As shown in **Supplementary Figure S3**, **Figures 5B, C**, the high stromal, immune, and estimate scores were associated with DLBCL patients with a high level of *S100A8* in both GSE87371 (31) and NCICCR_DLBCL (1) datasets. Meanwhile, there was not a significant difference (*P*>0.05) between high- and low- estimate scores both two datasets (**Supplementary Figures S3G, H**). On the other hand, the CIBERSORT algorithm (53), xCell algorithm (54), quanTIseq algorithm (55), and MCPcounter algorithm (41) were used to estimate the distribution and proportion of macrophages, CD8 T cells, and B cells. When combined with all selected algorithms, the significantly decreased abundance of macrophages M1, CD8 T cells were associated with high *S100A8* expression, and B cells expressed the opposite trend (**Figures 6B, C**). There were 79 ARG enrolled (**Supplementary Table S3**), and the positive correlation between S100A8 expression and the expression levels of IL1RN, IL1B, CXCL8, TYMP, PTGS2, CCL2, HGF, SOD2, NRP2, THBS1, ITGAV, and TIMEP2 (**Supplementary Figures S3I, J**) were investigated, indicating S100A8 might involve into the regulation of angiogenesis in TME.

### Prediction of potential anti-cancer compounds

**Figure 7:**
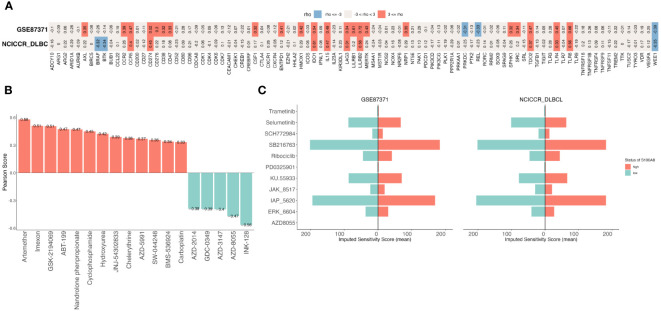
The Prediction of treatment responses of DLBCL patients. **(A)** The correlation between S100A8 and immune checkpoints in GSE87371 and NCICCR_DLBCL datasets. **(B)** The drug sensitivity analysis was showed that a total of 18 drugs were explored though CellMiner database. **(C)** The exploration of drugs through oncoPredict package, there were eleven drugs both GSE87371 and NCICCR_DLBCL datasets. DLBCL, Diffuse large B-cell lymphoma; GSE, Gene Expression Omnibus Series.

Immune checkpoint inhibitors can block checkpoint proteins or their partner proteins so that immune cells called T cells recognize and kill tumors (44, 56). We downloaded a list of the immune checkpoint targets from CKTTD (44), The correlation between the S100A8 expression and the immune checkpoint targets was calculated (**Figure 7A**). The result showed that S100A8 were positively correlated with CCR5, CD274(PD-1), IDO1, IL15, LAG3, LILRB2, TDO2, TLR4, and TLR8, while there was a negative correlation with WEE1 in both GSE87371 (31) and NCICCR_DLBCL (1) datasets. Based on the CellMiner database (45), the relationship between S100A8 expression and drug response was explored (**Figure 7B**). We observed that S100A8 expression was negatively associated with AZD-2014, GDC-0349, AZD-3147, AZD-8055, and INK-128. While a positive correlation with Artemether, Imexon, GSK-2194069, ABT-199, Nandrolone phenpropionate, Cyclophosphamide, Hydroxyurea, JNJ-54302833, Chelerythrine, AZD-5991, SW-044248, BMS-536924, Carboplatin and S100A8 expression was identified. On the other hand, we assessed the drug therapeutic responses between high- and low-S100A8 levels in both GSE87371 (31) and NCICCR_DLBCL (1) datasets using oncoPredict packages (46). Fourteen drugs and twenty-three drugs were explored in GSE87371 (31) and NCICCR_DLBCL (1) datasets, respectively (**Supplementary Figure S4**). There were eleven drugs that Selumetinib, IAP_5620, SB216763, ERK_6604, KU.55933, JAK_8517, SCH772984, Ribociclib, Trametinib, PD0325901, and AZD8055 were considered potential sensitivity small molecule inhibitors for both of them (**Figure 7C**).

## Discussion

Currently, S100A8 plays complex and multifaceted roles, with both tumor inhibition and promotion functions depending on the different tumor types (15, 16). Overexpression of S100A8 in breast cancer (57), ovarian cancer (58), liver cancer (59), acute myeloid leukemia (60), and melanoma (12), suggests its increased expression has been associated with tumor growth, invasion, and metastasis (16, 28). A total of 735 cancer patients enrolled in 5 studies indicated a negative association between the expression of S100A8 and poor disease-free survival (DFS) (23). On the other hand, upregulated S100A8 was correlated with a better prognosis in all gastric cancer patients (61). Due to this complexity, the role of S100A8 in tumors needs to be investigated and evaluated carefully, taking into account the broader context of cancer biology. In this study, we identified the gene expression profiles of public DLBCL datasets horizontally to find for the first time that elevated S100A8 expression is correlated with poor survival in DLBCL.

Mechanically, S100A8 can promote the inflammatory response by identifying the function enrichment of DEGs between high and low expression of S100A8 in DLBCL, and S100A8 also performed as a potent regulator of inflammation in the invasion and metastasis of multiple tumors (14, 15, 19). In this study, the IL-17 signaling pathway is identified as one of the most important molecular mechanisms in S100A8. Meanwhile, upregulation of S100A8 was verified in our cohort and *in vitro* experiments. By exposure to Paquinimod, which is considered a specific S100A8 inhibitor (49, 50), the expression of S100A8 was downregulated and associated with increased apoptosis, which evidently suggests that inhibition of S100A8 might induce cell apoptosis and increase DLBCL cell death. S100A8/S100A9 can suppress neutrophil apoptosis by inducing the phosphorylation of AKT, ERK, p38 MAPK, and JNK, as well as activating NF-κB in bronchial epithelial cells (62). The sensitivity of leukemia cells to chemotherapy and apoptosis should be increased through S100A8 silencing (60, 63), and the potential mechanism is related to the increased intracellular Ca2+ levels and apoptosis induced by endoplasmic reticulum stress (63). On the contrary, after treatment of S100A8/S100A9 in some tumor cells, it can induce cell death through apoptosis and autophagy, and this process would be rectified using N-acetyl-L-cysteine (NAC) through decreasing mitochondria reactive oxygen species (ROS) (64). Above all, these evidences demonstrated that the cell death regulated by S100A8 is full of complexity and diversity. We suggested that S100A8 inhibitors might promote apoptosis through a BCL-2-mediated signaling pathway in DLBCL.

Essentially, the structures, localization, and TME of S100A8 are related to the complex capacity of various functions (14, 16–18). In this study, S100A8 was enriched mainly in differential cell types, specifically macrophages, through scRNA-seq analysis using a public dataset, which contributed to the specific inflammatory milieu (19). Meanwhile, the S100A8 expression was positively associated with ESTIMATE scores in DLBCL in this study. Generally, ESTIMATE scores that included stromal and immune cells could predict tumor purity. A high ESTIMATE score can indicate an abundance of immune cells, including lymphocytes and macrophages, at the tumor site (65). It may also indicate a state of chronic inflammation and immune system dysregulation, which can promote tumor progression and treatment resistance. Recently, immune system dysregulation might contribute to the pathogenesis of DLBCL (66). Innate immune cells, such as macrophages, neutrophils, dendritic cells, and natural killer (NK) cells, may induce inflammation that contributes to the anti-tumor function or carcinogenic effect under abnormal immunity (67–69). The TME is an important aspect in the assessment of progression and the novel immunotherapeutic strategies of DLBCL (9, 10). Serna et al. found higher proportions of resting M0 and pro-inflammatory M1 macrophages in DLBCL than spleen samples (69), suggesting uncontrolled macrophages might disturb TME in DLBCL. Previous studies revealed that several derived genetic alterations might impact the immune recognition of TME formation (43, 70). S100A8 could induce a pro-inflammatory response, including inflammatory cytokines and chemokines, and introduce the tumor-promoting effect and suppression of anti-tumor immune responses (14, 19). Tumor-associated macrophages (TAMs) can have a spectrum of phenotypes and be associated with a poor prognosis (71), A meta-analysis demonstrated that the high abundance of M2 TAMs in TME might be related to an adverse clinical outcome for DLBCL patients (72). The interaction with S100A8 can influence several aspects of tumor biology, which is associated with immunosuppression and TME remodeling through driving the polarization of macrophages toward an M2-like phenotype. Angiogenesis interacted with TME within DLBCL to impact the development and progression of cancer. S100A8 might activate Toll-like receptor 4 (TLR4), which is involved in immune responses (14, 73) and leads to the release of pro-inflammatory cytokines and growth factors that contribute to angiogenesis (74). Also, the receptor for advanced glycation end products (RAGE) that is implicated in promoting angiogenesis can be linked to S100A8. Our results demonstrated that the S100A8 level shared the same trend as some ARGs, though analysis of GSE87371 and NCICCR_DLBCL, in particular, IL1RN, IL1B, and CXCL8 were also DEGs between high and low expression of S100A8. It showed that the release of some angiogenic factors might be infected by S100A8, and these factors play an effective role in promoting angiogenesis by triggering endothelial cell proliferation and migration (75). Synthetically, S100A8 may interact with some regulators within the TME, contributing to inflammation, tumorigenesis, and angiogenesis.

Subsequently, the correlation between immune checkpoints, which play a crucial role in the regulation of the immune system, and S100A8 expression was investigated. Increased evidence has demonstrated that immune checkpoint inhibitors (ICIs) are a revolutionary approach in tumor treatment by enhancing the natural immune response against tumors (76, 77). Interestingly, a positive correlation between CCR5, PD-1, IDO1, IL15, LAG3, LILRB2, TDO2, TLR4, and TLR8 expression and S100A8 expression was found. Specifically, PD-1, a common immunosuppressive member on T cells, indicates T cell exhaustion and plays an important role in hindering the ability of anti-cancer (78). Similarily, high levels of S100A8 that also exist in TME might potentially contribute to this immunosuppressive environment (19, 28). Recent studies (19, 79) have raised the possibility that S100A8 might link some immune checkpoints to play an immunosuppressive role in the occurrence and development of tumors. These findings suggested that S100A8 had potential for immunotherapy in DLBCL. We also analyzed the IC50 of different drugs and found that S100A8 expression patients respond to these drugs. Based on the training and validation datasets, a total of 11 drugs were explored. Among them, Selumetinib, Ribociclib, and Trametinib have been in clinical use. Selumetinib, as a MEK inhibitor, introduced dose-dependent apoptosis in DLBCL cell lines and suppressed tumor growth in xenograft models (80). Trametinib is also a MEK inhibitor approved both as a single agent and in combination with other chemotherapeutic drugs for the treatment of metastatic melanoma (81). Ribociclib has been used to treat a variety of tumors through inhibition of CDK4/6 (82). *In vitro* experiments, Paquinimod has been proven to inhibit S100A8 in a dose-dependent manner (49, 50). S100A8/S100A9 plays a role in activating TLR4-ERK1/2-Drp1-dependent mitochondrial fission and dysfunction in mediating septic cardiomyopathy. Administration of Paquinimod should prevent mitochondrial dysfunction in septic cardiomyopathy through inhibition of S100A8/S100A9 (83). Interestingly, Paquinimod effectively alleviated pneumonia by reducing viral loads in mice infected with SARS-CoV-2 (50). Above all, we suggested these drugs would be novel references in the future treatment of DLBCL.

Simultaneously, cell adhesion-mediated drug resistance (CAM-DR) is a phenomenon where cancer cells, through interactions with the extracellular matrix (ECM) and neighboring cells, gain resistance to chemotherapy (84, 85). Increasing studies (85, 86) demonstrated that CAM-DR plays a crucial role in the relapse and death of NHL patients, and inhibition of this cell adhesion-mediated signal should improve the therapeutic sensitivity of tumor cells. Tjin et al. reported that activation of HGF/MET signaling could induce integrin-mediated adhesion to lymphomagenesis in DLBCL (87). High expression of ABCG2 within the stromal microenvironment in DLBCL correlated inversely with survival, which could alleviate the stroma-induced chemotolerance through inhibition of ABCG2 level (88). Thus, the study on the effect of S100A8 on CAM-DR is lacking. S100A8 has been associated with regulation of the TME, potentially influencing the adhesive properties of tumor cells. Loss of S100A8/S100A9 might lead to dysregulated integrin-mediated adhesion and migration (89), and integrins are cell surface receptors involved in cell adhesion. We have reason to believe that targeting S100A8 should improve tumor resistance, especially in the context of CAM-DR, and a new study should be investigated in the future.

Additionally, there were some limits to this study. First, we enrolled the public datasets and our retrospective cohort, and the generalizability was limited. Prospective validations should be carried out. Second, the effect of S100A8 in DLBCL was explored through *in vitro* experiments; the effect on the influence of apoptosis through inhibition of S100A8 using Paquinimod, lacking the trials through knocking down S100A8 directly, and *in vivo* experiments are lacking for this analysis. Third, due to the complexity and diversity of S100A8 in TME, we did not verify our findings at the transcriptional level because there were no fresh or fresh frozen samples.

## Conclusion

Summarily, this study is the first to intensively illustrate the role of S100A8 in DLBCL. We identified S100A8 as the positive diagnostic and prognostic biomarker for DLBCL. Inhibition of S100A8 can promote cell apoptosis and suppress tumor growth. S100A8 is related to the regulation of immune responses and has revealed its potential for predicting immunotherapy responses, offering novel ideas for the administration of ICIs in DLBCL.

## Data availability statement

The original contributions presented in the study are included in the article/**Supplementary Material**. Further inquiries can be directed to the corresponding author.

## Ethics statement

The studies involving humans were approved by the Affiliated Hospital of Putian University’s Ethics Committee. The studies were conducted in accordance with the local legislation and institutional requirements. The participants provided their written informed consent to participate in this study. The manuscript presents research on animals that do not require ethical approval for their study.

## Author contributions

QL: Investigation, Methodology, Validation, Visualization, Writing – original draft. JS: Methodology, Writing – original draft. YF: Data curation, Funding acquisition, Investigation, Resources, Writing – original draft. ZZ: Data curation, Methodology, Writing – original draft. JC: Methodology, Writing – original draft. CZ: Conceptualization, Funding acquisition, Writing – review & editing.
