# Supplementary material for: S100A8 is a prognostic signature and associated with immune response in diffuse large B-cell lymphoma
Source: Front Oncol. 2024 Feb 1;14:1344669. doi: 10.3389/fonc.2024.1344669 (PMC10867108; doi:10.3389/fonc.2024.1344669)
Supplement: Supplementary file 1 [file DataSheet_1.pdf]

Supplementary Material

1 Supplementary Figures and Tables

1.1 Supplementary Figures

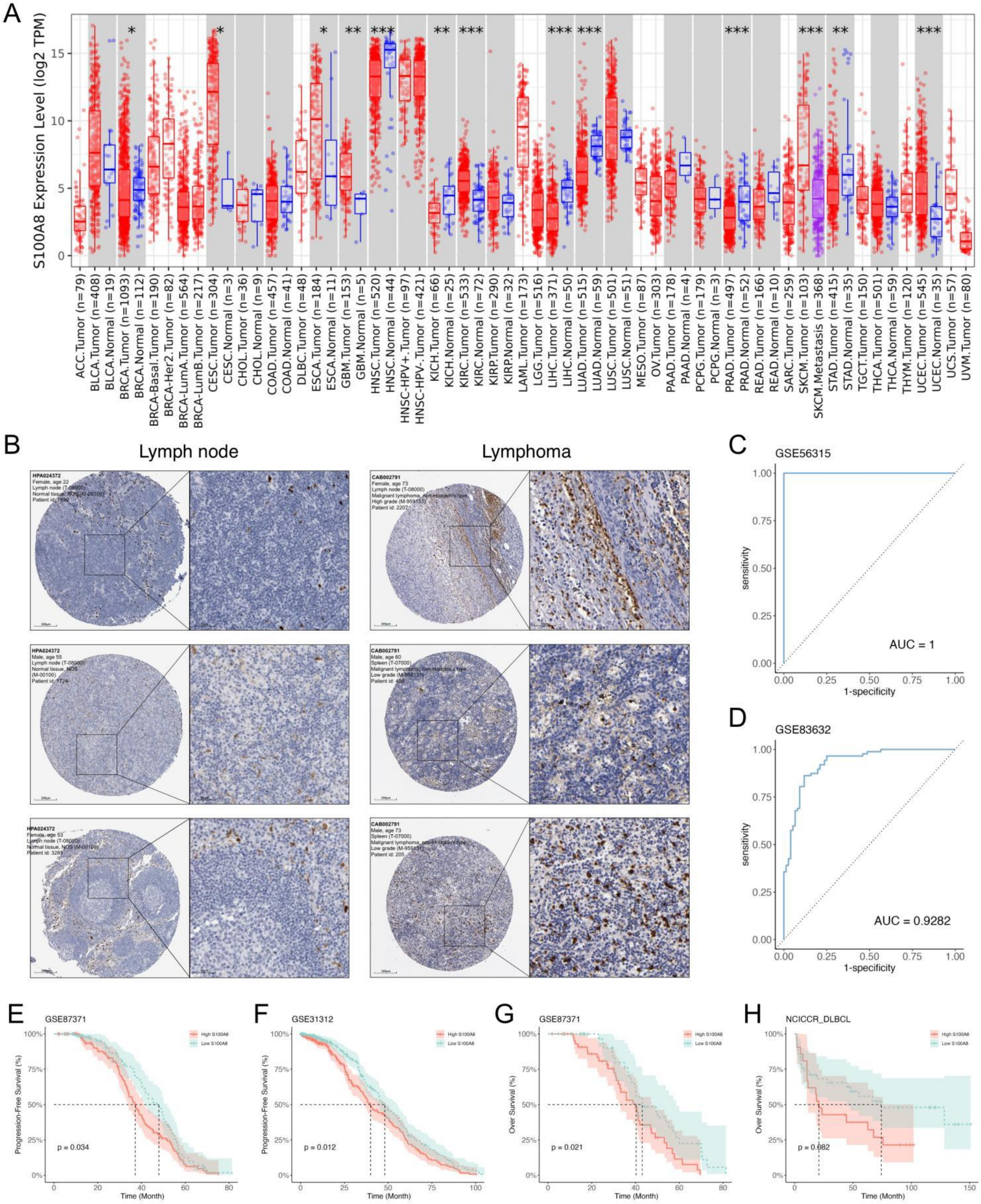

Supplementary Figure S1. The upregulated expression of S100A8 in Lymphoma. **A.** The gene expression profile of S100A8 in different types of tumors and its homologous normal tissues using

TCGA data through TIMER2.0 (http://timer.cistrome.org). **B.** S100A8 expression in lymphoma tissue and lymph node through The Human Protein Altas (HPA, https://www.proteinatlas.org). The ROC curves and AUC for evaluating the prediction accuracy of S100A8 in the network analysis of GSE56315 (**C**) and GSE83632 (**D**) datasets. The KM curves showed PFS based on GSE87371 (**E**) and GSE31312 (**F**) datasets. Meanwhile, the KM curves showed OS on different IPI group (IPI > 2) based on GSE87371 (**G**) and NCICCR_DLBCL (**H**) datasets. DLBCL, Diffuse large B-cell lymphoma; GSE, Gene Expression Omnibus Series; IPI, international prognostic index; KM, Kaplan–Meier; PFS, Progression-Free survival.

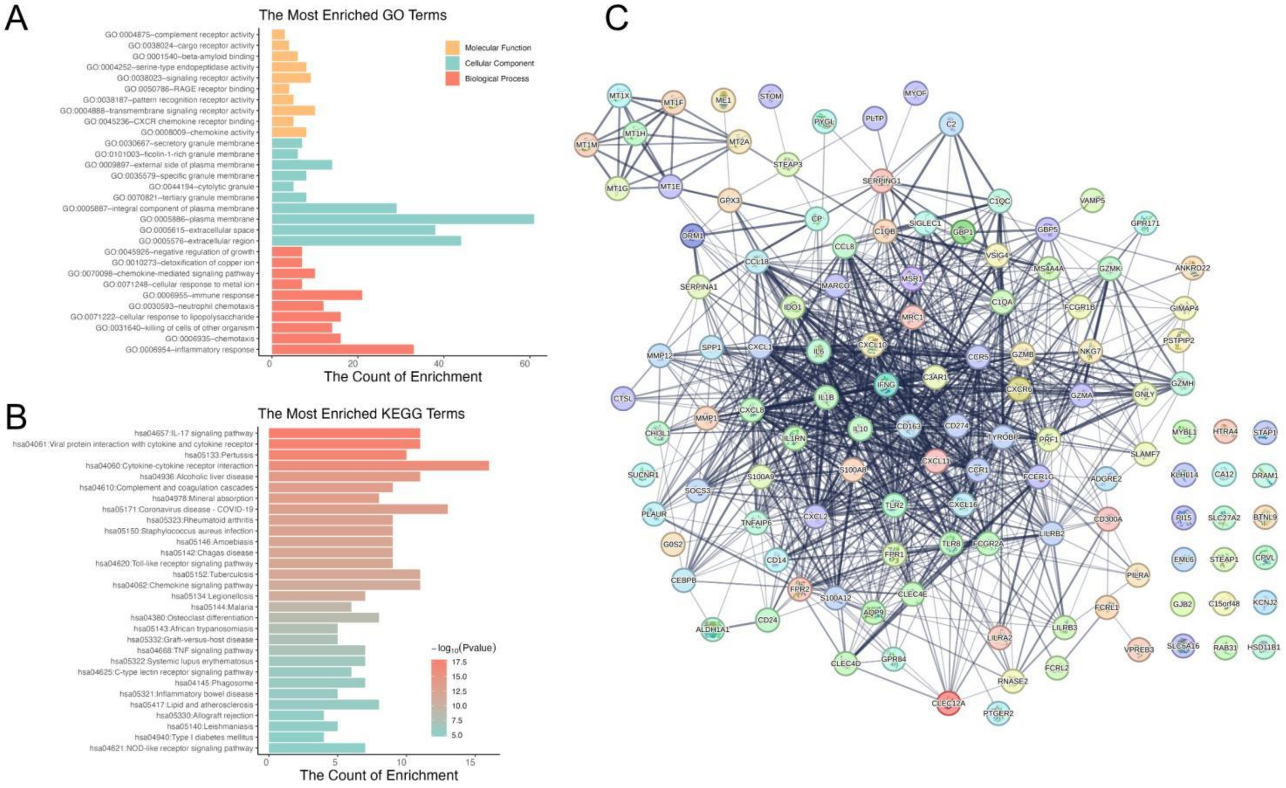

Supplementary Figure S2. The function enrichment of S100A8 in DLBCL. **A.** GO enrichment analysis of 123 genes enrolled. **B.** KEGG pathway analysis of 123 genes enrolled (top 30). **C.** The PPI network of 123 genes enrolled in DLBCL patients visualized by the STRING website (https://string-db.org). DLBCL, Diffuse large B-cell lymphoma; GO, Gene Ontology; GSE, Gene Expression Omnibus Series; KEGG, Kyoto Encyclopedia of Genes and Genomes; PPI, protein-protein interaction; STRING, Search Tool for the Retrieval of Interacting Genes/Proteins.

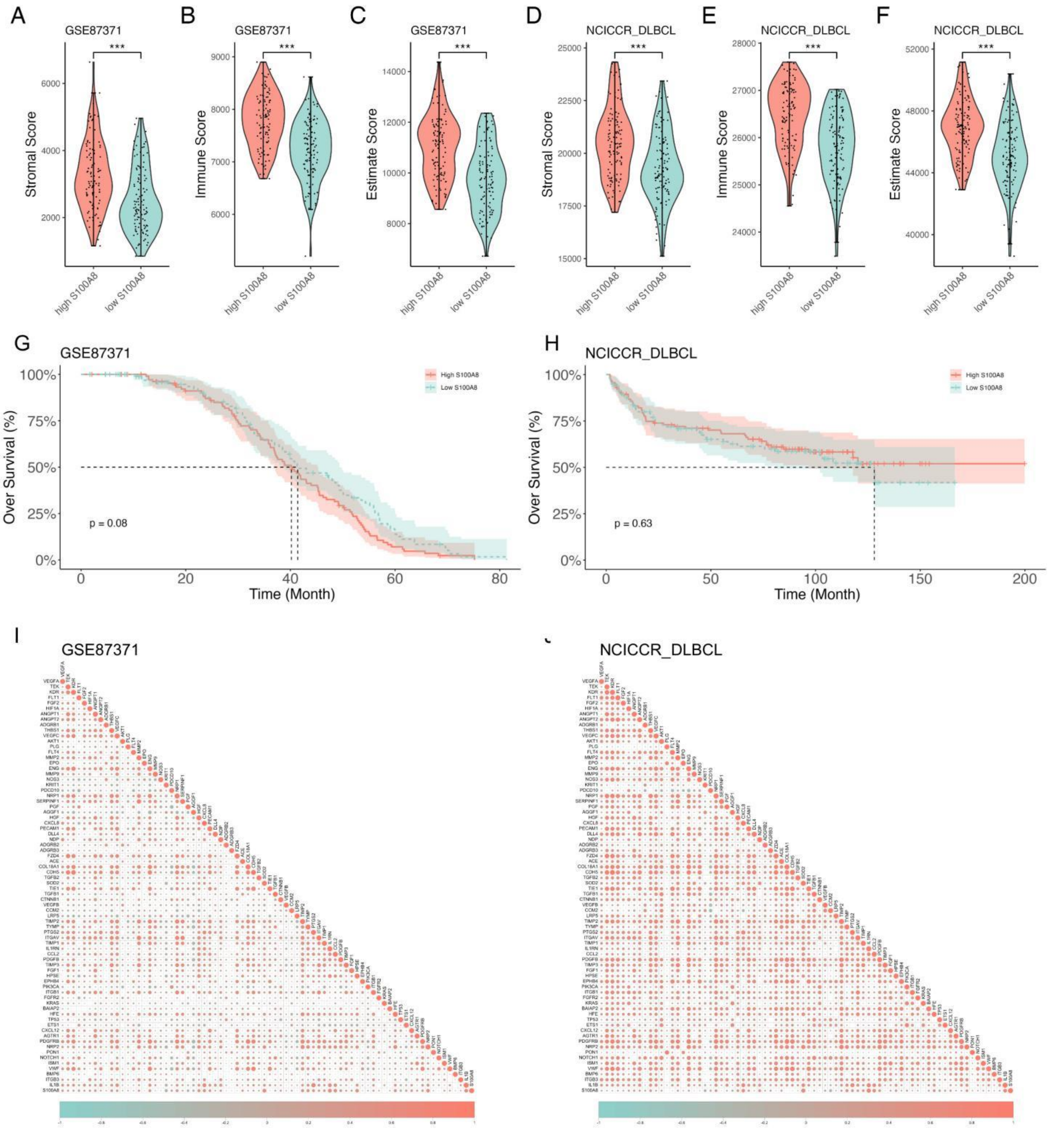

Supplementary Figure S3. The The immune infiltration associations of S100A8 in GSE87371 and NCICCR_DLBCL. **A. and D.** Comparison between the stromal scores of high and low S100A8; **B. and E.** Comparison between the immune scores of high and low S100A8; **C. and F.** Comparison between the ESTIMATE scores of high and low S100A8. The KM curves showed OS compared with high and low ESTIMATE scores based on GSE87371 (**G**) and NCICCR_DLBCL (**H**) datasets. (**I, J**). The correlation analysis between ARG and the expression of S100A8. ARG, angiogenesis-related genes; DLBCL, Diffuse large B-cell lymphoma; GSE, Gene Expression Omnibus Series; KM, Kaplan–Meier; OS, over survival.

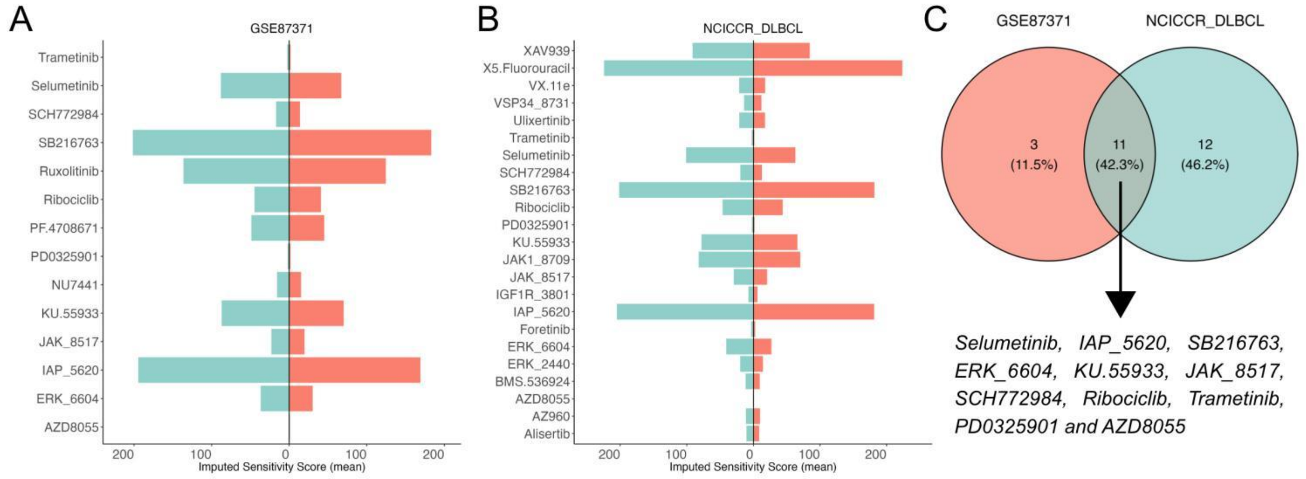

Supplementary Figure S4. The Prediction of treatment responses of DLBCL patients in GSE87371 and NCICCR_DLBCL. **A.** The exploration of drugs based on GSE87371 through oncoPredict package. **B.** The exploration of drugs based on NCICCR_DLBCL through oncoPredict package. **C.** Eleven drugs were intersected from GSE87371 and NCICCR_DLBCL datasets. DLBCL, Diffuse large B-cell lymphoma; GSE, Gene Expression Omnibus Series.

1.2 Supplementary Tables

Supplementary Tables S1. Clinical data of our cohort enrolled in this study

Parameters	DLBCL	RHL
Number	25	14
Age	62.60±12.53	40.64±16.02
Gender(Male)	20	5
COO(GCB)	13	0
IPI(<=2)	15	0

Supplementary Tables S2. Primer of sequences

GENE SYMBOL	PRIMER (5' - 3')
S100A8	F: TGCTAGAGACCGAGTGTCCT
	R: GCCACGCCCATCTTTATCAC
β-actin	F: TGACGTGGACATCCGCAAAG
	R: CTGGAAGGTGGACAGCGAGG

Supplementary Tables S3. 79 angiogenesis-related genes retrieved from the GeneCards (Score >10)

Symbol	Description
VEGFA	Vascular Endothelial Growth Factor A
TEK	TEK Receptor Tyrosine Kinase
KDR	Kinase Insert Domain Receptor
FLT1	Fms Related Receptor Tyrosine Kinase 1
FGF2	Fibroblast Growth Factor 2
HIF1A	Hypoxia Inducible Factor 1 Subunit Alpha
ANGPT1	Angiopoietin 1
ANGPT2	Angiopoietin 2
ADGRB1	Adhesion G Protein-Coupled Receptor B1
THBS1	Thrombospondin 1
VEGFC	Vascular Endothelial Growth Factor C
AKT1	AKT Serine/Threonine Kinase 1
PLG	Plasminogen
FLT4	Fms Related Receptor Tyrosine Kinase 4
MMP2	Matrix Metallopeptidase 2
EPO	Erythropoietin
ENG	Endoglin
MMP9	Matrix Metallopeptidase 9
NOS3	Nitric Oxide Synthase 3
KRIT1	KRIT1 Ankyrin Repeat Containing
PDCD10	Programmed Cell Death 10
NRP1	Neuropilin 1
SERPINF1	Serpin Family F Member 1
PGF	Placental Growth Factor
AGGF1	Angiogenic Factor With G-Patch And FHA Domains 1
HGF	Hepatocyte Growth Factor
CXCL8	C-X-C Motif Chemokine Ligand 8
PECAM1	Platelet And Endothelial Cell Adhesion Molecule 1
DLL4	Delta Like Canonical Notch Ligand 4
NDP	Norrin Cystine Knot Growth Factor NDP
ADGRB2	Adhesion G Protein-Coupled Receptor B2
ADGRB3	Adhesion G Protein-Coupled Receptor B3
FZD4	Frizzled Class Receptor 4
ACE	Angiotensin I Converting Enzyme
COL18A1	Collagen Type XVIII Alpha 1 Chain
CDH5	Cadherin 5
TGFB2	Transforming Growth Factor Beta 2
SOD2	Superoxide Dismutase 2
MIR21	MicroRNA 21
TIE1	Tyrosine Kinase With Immunoglobulin Like And EGF Like Domains 1
VEGFD	Vascular Endothelial Growth Factor D
TGFB1	Transforming Growth Factor Beta 1
CTNNB1	Catenin Beta 1
VEGFB	Vascular Endothelial Growth Factor B
CCM2	CCM2 Scaffold Protein

Symbol	Description
LRP5	LDL Receptor Related Protein 5
TIMP2	TIMP Metallopeptidase Inhibitor 2
TYMP	Thymidine Phosphorylase
PTGS2	Prostaglandin-Endoperoxide Synthase 2
ITGAV	Integrin Subunit Alpha V
TIMP1	TIMP Metallopeptidase Inhibitor 1
VTN	Vitronectin
IL1RN	Interleukin 1 Receptor Antagonist
CCL2	C-C Motif Chemokine Ligand 2
PDGFB	Platelet Derived Growth Factor Subunit B
TIMP3	TIMP Metallopeptidase Inhibitor 3
FGF1	Fibroblast Growth Factor 1
HPSE	Heparanase
EPHB4	EPH Receptor B4
PIK3CA	Phosphatidylinositol-4,5-Bisphosphate 3-Kinase Catalytic Subunit Alpha
ITGB1	Integrin Subunit Beta 1
FGFR2	Fibroblast Growth Factor Receptor 2
CCN2	Cellular Communication Network Factor 2
KRAS	KRAS Proto-Oncogene, GTPase
BAIAP2	BAR/IMD Domain Containing Adaptor Protein 2
HFE	Homeostatic Iron Regulator
TP53	Tumor Protein P53
ETS1	ETS Proto-Oncogene 1, Transcription Factor
CXCL12	C-X-C Motif Chemokine Ligand 12
AGTR1	Angiotensin II Receptor Type 1
PDGFRB	Platelet Derived Growth Factor Receptor Beta
NRP2	Neuropilin 2
PON1	Paraoxonase 1
NOTCH1	Notch Receptor 1
ISM1	Isthmin 1
VWF	Von Willebrand Factor
BMP6	Bone Morphogenetic Protein 6
ITGB3	Integrin Subunit Beta 3
IL1B	Interleukin 1 Beta
